# A Unique Case of Harlequin Ichthyosis in the Tertiary Health Care System in a Rural Area

**DOI:** 10.7759/cureus.43342

**Published:** 2023-08-11

**Authors:** Aashka C Lainingwala, Sahini Gajula, Umaima Fatima, Sabah Afroze, Sarojini Posani, Mudit Moondra, Nisarg P Mangukiya, Mihirkumar P Parmar, Vishal Venugopal

**Affiliations:** 1 Internal Medicine, Gujarat Medical Education & Research Society Medical College, Vadnagar, Mehsana, IND; 2 Internal Medicine, Gandhi Medical College and Hospital, Secunderabad, IND; 3 Internal Medicine, Shadan Institute of Medical Sciences, Teaching Hospital and Research Centre, Hyderabad, IND; 4 Internal Medicine, Sri Devaraj Urs Medical College, Kolar, IND; 5 Internal Medicine, Rabindranath Tagore Medical College, Udaipur, IND; 6 Internal Medicine, Bhaarath Medical College & Hospital, Chennai, IND

**Keywords:** pre-term, abca12 gene mutation, genetic skin disorders, congenital ichthyosis, harlequin ichthyosis

## Abstract

Harlequin ichthyosis (HI) is a severe and rare genetic anomaly that affects skin development and leads to the formation of thick, diamond-shaped plates of keratinized skin. The adenosine triphosphate binding cassette A 12 (ABCA12) gene, which is essential for the transportation of lipids required for the skin's barrier function, has mutations that result in this condition. The affected individuals exhibit distinct clinical features, including thickened skin, deep cracks, and fissures, which can result in significant physical and functional impairments. HI is usually apparent at birth, with affected infants presenting with tight and rigid skin that restricts movement and normal growth. The condition is associated with various complications, including difficulty breathing, feeding difficulties, and increased susceptibility to infections. Due to the impaired skin barrier, affected individuals are also prone to dehydration and temperature dysregulation. In this case report, we present a unique case of ichthyosis in a nine-month-old child. Despite advances in medical care, HI remains a challenging condition with a high mortality rate, particularly in the neonatal period. However, with early detection, appropriate interventions, and an improved understanding of the underlying molecular mechanisms, there is hope for enhanced management and improved quality of life for individuals living with HI.

## Introduction

Harlequin ichthyosis (HI) is a rare genetic disorder with an incidence ranging from 1/300000 to 1/1000000 worldwide [1]. HI is inherited in an autosomal recessive manner (5P, 9P) and is brought on by mutations in the adenosine triphosphate binding cassette A 12 (ABCA12) gene. In a large proportion of chromosomes from HI children, an ABCA12 gene mutation has been found [2]. The protective lipid layer on the skin is destroyed as a result of a flaw in the cell's lipid transporter, leaving the youngster more vulnerable to infections and heat loss. Everywhere on the body, HI is characterized by thick, plaque-like scales and is linked to ectropion, eclabium, and ear abnormalities; sparse scalp hair; hypoplastic digits; and a total lack of eyebrows and eyelashes. Babies who have lost their lipid layer are at a higher risk for seizures, skin and respiratory infections, dehydration, low salt, poor nutrition, and low body temperature. The mortality rate is quite high in these situations as a result of the skin's faulty barrier function [3]. Some ichthyosis newborns live for several months or even years [4]. A multidisciplinary team, involving nursing staff, physical therapy experts, orthopedic specialists, plastic surgeons, ophthalmologists, otolaryngologists, geneticists, dermatologists, and neonatologists, is required to manage HI [5].

However, nursing personnel play an important role in management by preventing infection. Here, we are presenting a unique case of HI that was delivered at 36 weeks and died after nine days of life.

## Case presentation

A 36-week-old baby was presented in the neonatal intensive care unit after spontaneous normal vaginal delivery because of the unusual appearance of the baby, which was not diagnosed earlier in any antenatal ultrasonography. The baby cried immediately after birth and had a low birth weight of around 1.80 kilograms. The APGAR (Appearance, Pulse, Grimace, Activity, and Respiration) score was 8 out of 10 at one minute, and it is 9 out of 10 at five minutes, which was in the normal range. Physical examination showed a pulse rate of around 140/min on the radial artery at the baby’s wrist, a respiratory rate of around 62/min, and oxygen saturation (SpO2) was around 96% on the nasal cannula. Bluish discoloration of the skin (cyanosis) was present on peripheral areas like the hands, fingertips, and toes. Both parents reported having a consanguineous marriage, and her antenatal history suggested that her age at conception was around 20 years old, and this is the first case in the family.

Antenatal ultrasonography was done at two and four months. Fetal cardiac activity and fetal movements were present on antenatal ultrasonography at two months, and liquor was adequate. At four months, an antenatal ultrasound showed fetal cardiac activity and fetal movements, as well as a floating presentation and liquor that was adequate. 3D ultrasonography for anomalies was not done when the mother was admitted to the obstetrics ward with a history of labor pain. During the abdominal examination, the uterus was measured as 34 weeks long, was cephalic, and fetal heart sounds were present. A vaginal examination revealed that OS is two fingers loose, blood pressure was around 136/98 mmHg on the right brachial artery at the right arm with a sphygmomanometer, and the mother was reportedly having preeclampsia at that time, so injections of Tidilan, which is a vasodilator and uterine relaxant, and tablets containing labetalol (Lobet), which is an alpha and beta-adrenergic antagonist, were given to control the preeclampsia.

The reports of the mother are listed in Table 1.

**Table 1:** Hematology and biochemistry report RBC: red blood cells, MCV: mean corpuscular volume, MCH: mean corpuscular hemoglobin, RDWcv: red cell distribution width coefficient of variation, WBC: white blood cells

TESTS	RESULTS	REFERENCE RANGE
BLOOD COUNTS AND INDICES		
HEMOGLOBIN	11.50 gm%	13-17 gm%
TOTAL RBC	3.67 mil/cmm	4.5-5.5 mil/cmm
MCV	94 fl	77-93 fl
MCH	31.50 pg	27-32 pg
RDWcv	11.60 %	11.6-14 %
TOTAL WBC	11200/cmm	4000-10000/cmm
PLATELET COUNT	218000/cmm	150000-450000/cmmq
Biochemistry Test		
SERUM NA+	136 mmol/L	137-145 mmol/L
SERUM BILIRUBIN	O.60 mg/dl	0.1-0.8 mg/dl
SERUM CREATININE	0.92 MG/DL	0.7-1.4 mg/dl
TOTAL PROTEIN	6 gm/dl	6.5-8.5 gm/dl
ALBUMIN	2.80 gm/dl	3.5-5.5 gm/dl
URIC ACID	7.14 mg/dl	15-45 mg/dl

**Figure 1:**
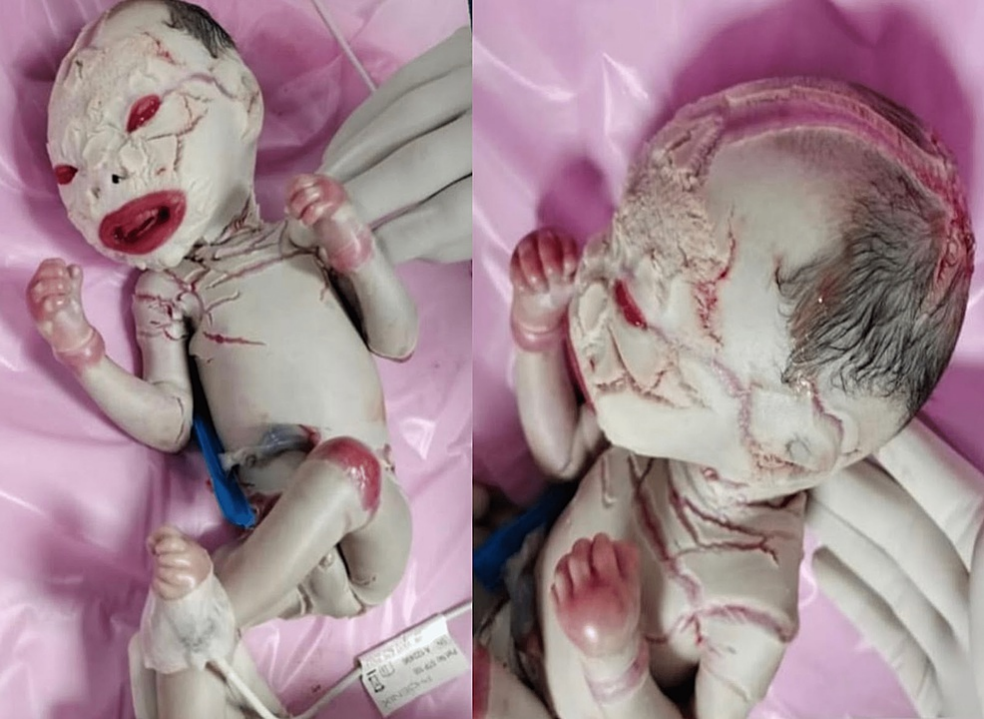
Day 1 of life Hands and feet were small, swollen, and partially flexed, and plantopalmar keratoderma was noted.

**Figure 2:**
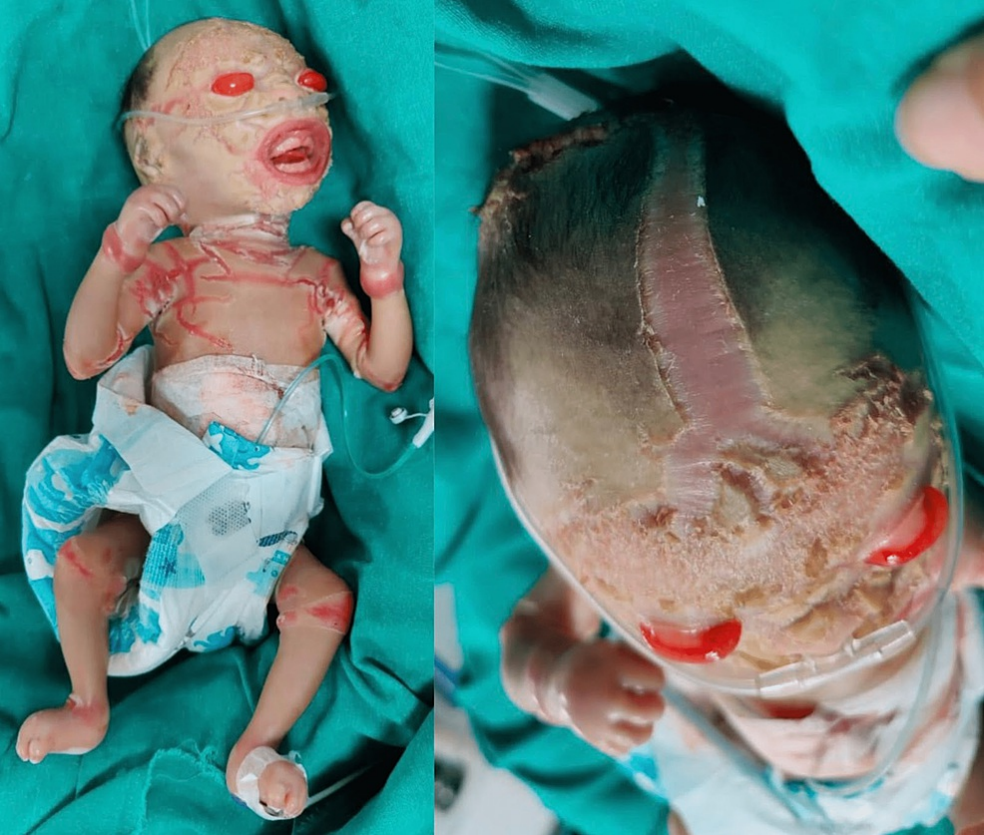
Day 4 of life The ears were unevenly shaped and fused to the head, and fontanels were poorly fused with marked alopecia.

The baby had severely scalded skin with deep fissures that covered most of the body parts and distorted facial features like tight skin around the eyes and mouth, which restricted the feeding ability of the baby and caused oral dryness (xerostomia). Outward turning of the lip (eclabium) and lower eyelid drooping away from the eye and turning outward (ectropion) were notably seen. Hyperkeratinization and severe inflammation of the skin surface (erythroderma) were seen on the majority of the body surface area, which restricts chest movements and caused respiratory distress. Electrolyte imbalance and dehydration were also seen and plantopalmar keratoderma was noted. Hands and feet were small, swollen, and partially flexed. The ears were unevenly shaped and fused to the head. Fontanels were poorly fused with marked alopecia (Figures 1, 2). So for management, the antibiotic 100 mg/kg piperacillin was given every eight hours, which helps prevent secondary infections and reduces the chances of sepsis. Oral retinoids 0.5 mg/kg/day were given at an early stage, which helps in increasing life expectancy. Petroleum jelly was also applied all over the body's surface area to help in the shedding of the hard skin. A nasal cannula was attached to help provide oxygen to the baby. Electrolyte imbalance was improved after intravenous infusion of dextrose in normal saline with a multivitamin pint and a ringer lactate pint two hours apart. Topical eye drops were used to prevent eye infections.

**Figure 3:**
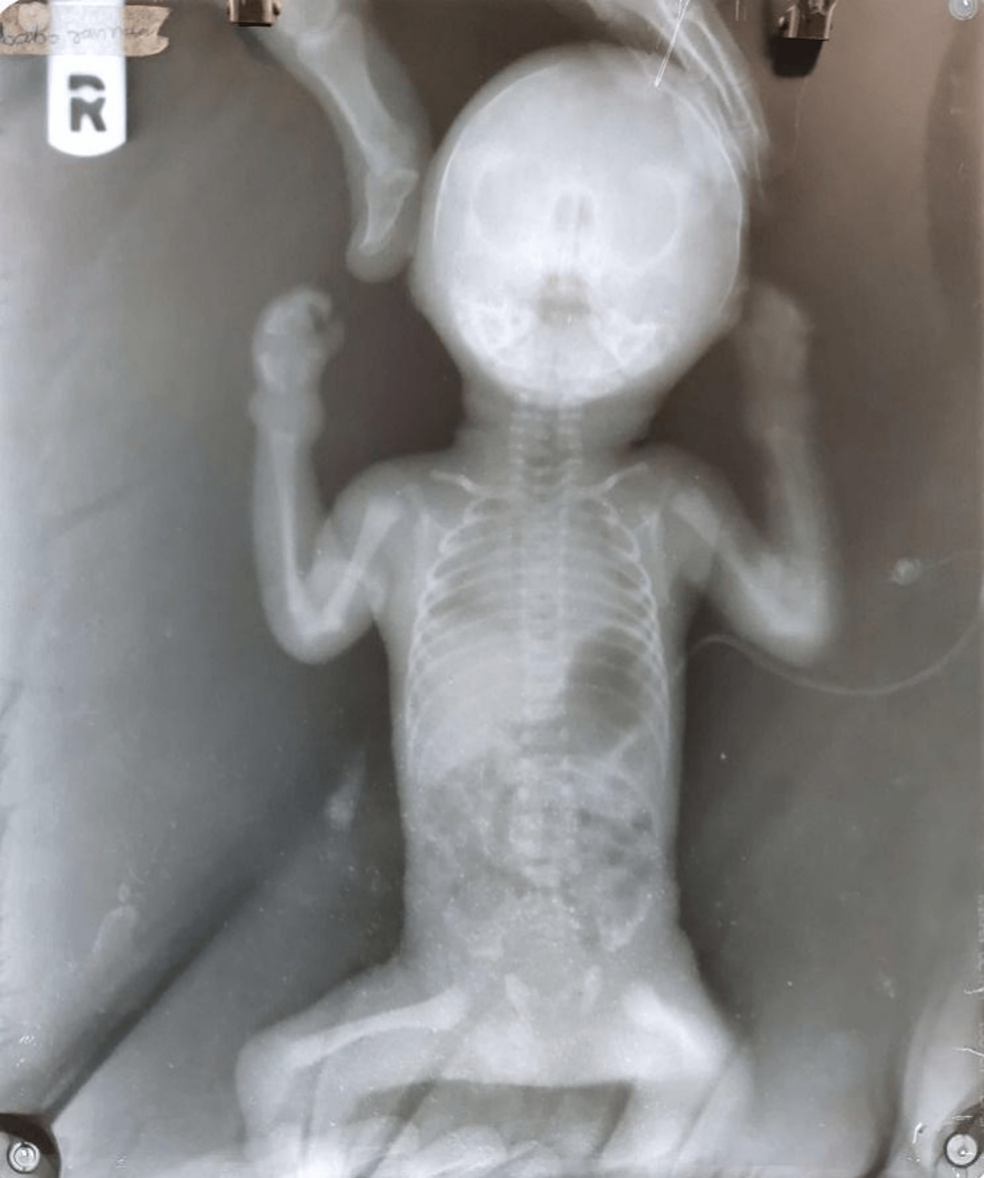
A radiographic X-ray of the baby Subluxation of joints was present.

A radiographic X-ray was also taken of the baby, which showed subluxation of the joints (Figure 3).

**Figure 4:**
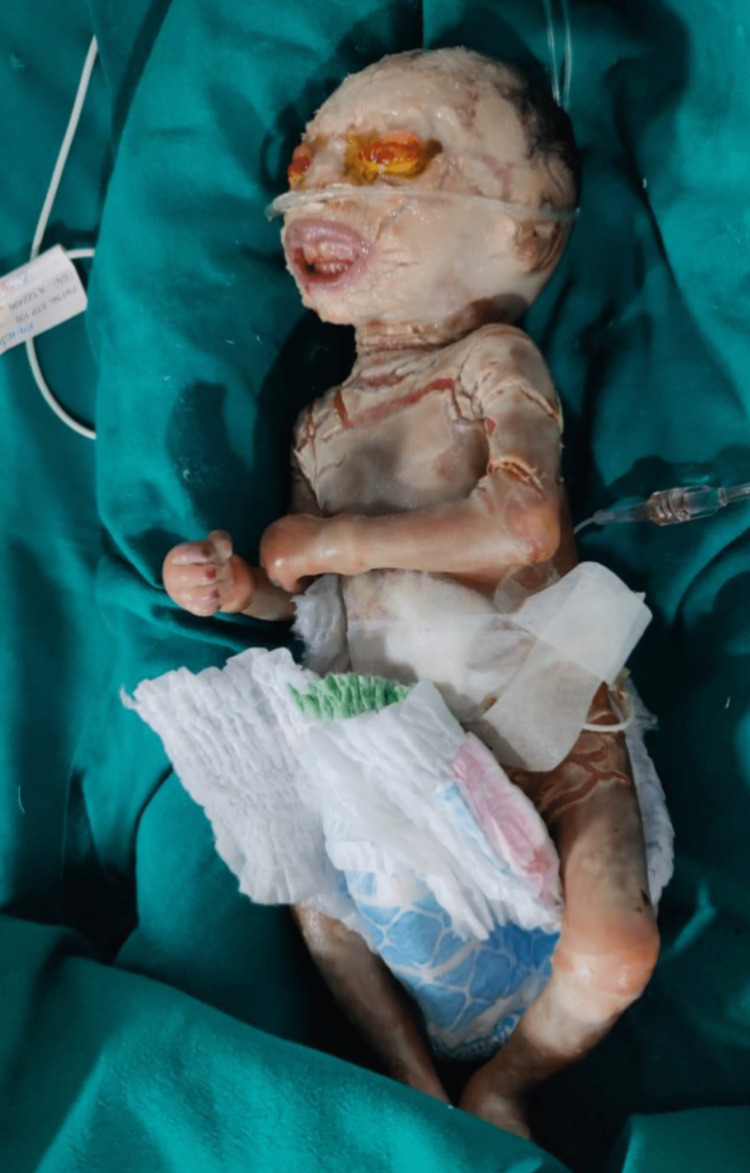
Day 7 of life The hard and thick skin started shedding off, and the baby had shown good improvement in limb movements.

A significant improvement was noted after the initial therapy (Figure 4). The hard and thick skin started shedding off, and the baby had shown good improvement in limb movements. but despite significant improvement, the baby died on the ninth day of life. Karyotyping and anomaly scanning were not done, and a skin biopsy was not taken. A diagnosis was made on the basis of a clinical examination showing scalded, tough skin with eclabium, ectropion, and hypertrophic skin changes. All of the clinical features were suggestive of autosomal recessive congenital ichthyosis.

## Discussion

HI is an uncommon and severe genetic anomaly of the skin that affects the developing fetus [5]. HI is a serious autosomal recessive genetic condition arising from a mutation in the ABCA12 gene, which codes for the adenosine triphosphate-binding cassette transporter, a cell membrane transporter involved in lipid transport [6]. This mutation causes faulty lipid production inside epidermal keratinocytes, leading to the loss of the skin lipid barrier and the development of HI in fetuses [5]. The disease affects one in every 300,000 live births [7]. The condition is extremely rare, and there is no proven treatment [8]. Infants with HI have a high mortality rate and a dismal prognosis, and the majority of neonates die shortly after birth from infection, heat loss, dehydration, electrolytic abnormalities, or respiratory distress [8]. Because of its severe clinical characteristics, HI was quickly identified at birth. But because of the development of prenatal diagnostic techniques, a number of HI cases with a family history were identified early on, and the families received sufficient counseling prior to the baby's birth [9]. A prenatal HI diagnosis can be made using tools like ultrasound. A number of HI characteristics, such as ectropion and eclabium, dysplastic ears, dysplastic ears, fixed hand positions, absent noses, edematous thighs and feet, fetal growth restriction, polyhydramnios, and skin with a "snowflake sign," might be seen by ultrasound [9].

Next-generation sequencing (NGS) is a technique for diagnosing uncommon genetic abnormalities such as congenital ichthyosis. This method is intended to detect causative mutations in sets of genes linked to one or more hereditary disorders [10]. Despite the fact that NGS is the most commonly used technology for identifying HI, clinical evaluation and ultrasound are required for an appropriate diagnosis. A prenatal diagnosis of an ABCA12 gene mutation can be made via DNA-based molecular testing on a chorionic villus sample or amniocentesis [11]. Although Harlequin ichthyosis cannot be prevented, it can be identified or diagnosed early in pregnancy. A diagnosis is made on clinical grounds. In this case, a 36-week-old preterm neonate with stable vitals and scalded, tough skin with eclabium, ectropion, and hypertrophic skin changes were suggestive of autosomal recessive congenital ichthyosis. Traditional prenatal screening in this case did not confirm the diagnosis of Harlequin ichthyosis. Although prenatal diagnosis for this condition is challenging, using DNA-based prenatal diagnosis has recently significantly improved this area of difficulty [12].

A prenatal diagnosis of HI may also be possible with the advancement of ultrasound imaging [5]. Three-dimensional ultrasound applications aid in the identification of facial physical characteristics, which substantially assists in prenatal diagnosis [13]. Consequently, it is important to consider genetic testing and counseling for parents who are at risk [9]. Infants with HI historically could not live through the newborn stage; however, recent developments in neonatal intensive care and coordinated multidisciplinary management have dramatically improved survival, and the prognosis of HI infants has significantly improved over the past 20 years [14,15]. Harlequin ichthyosis is frequently thought to be deadly, and treatment is typically palliative, but a follow-up study of 45 afflicted infants revealed that survival rates are increasing with proper neonatal care and the early introduction of oral retinoids [16], and for that, early systemic retinoids are widely used, although their use remains debatable [17]. Many cases of the disease are still being treated with supportive measures, which include early intubation and prevention of complications due to the skin barrier defect and its abnormality. Many patients require opioids for pain and interventions due to necrosis (digital necrosis is a common complication), and it is imperative to maintain optimal hydration, circulation, and kidney functions.

Intensive care is required for the majority of patients in the neonatal intensive care unit (NICU). A multidisciplinary team of professionals, including nurses, physical therapists, orthopedic experts, plastic surgeons, ophthalmologists, otolaryngologists, geneticists, dermatologists, and neonatologists, provides intensive care for the infant [17]. Direct intubation was necessary in the majority of cases due to the disease's high morbidity ratio and heightened risk of respiratory failure [17,18]. Immediate moisturizing of the baby can help soften the encasing membrane, enabling the child to move more freely and breathe deeply [19]. In 50% of the HI patients, respiratory failure was the only cause of death or a contributing factor, including 50% of those who passed away during the first three days of life. Along with the difficulties brought on by the infant's immature growth, electrolyte imbalance, respiratory distress, starvation, and infection should also be avoided [17,19].

Prophylactic antibiotics like piperacillin and retinoids are administered to prevent severe infections. Bilateral ectropion may develop, and infants are at a higher risk of developing strabismus, conjunctivitis, and keratitis. This complication is often treated with topical ophthalmic ointments like retinoids and sometimes reconstructive surgery [20]. For close observation, infant patients should be housed in a humidified isolette. Because impaired perspiration frequently results in overheating, it might be essential to keep the isolate at a slightly lower temperature than usual (32-34 °C) to maintain an ideal body temperature [19]. There aren't many case studies in the literature about the surgical management of HI. The knowledge gained from surgical cases is particularly useful in understanding digital ichthyosis, a prevalent HI symptom that jeopardizes the neurovascular integrity of the upper limbs. In situations of congenital constriction bands, circumferential release and subsequent Z-plasty restoration produce favorable results. Autologous skin grafts are currently the most popular surgical procedure. But because sepsis and bacterial infections in the epidermis and epidermal layer are very likely, invasive treatments need to be done with great care [17]. In addition to treating the illness, social workers and psychologists should provide parents with the necessary assistance. It is critical for the family to prepare for the prospect of losing their child because the majority of infants do not survive the neonatal period [21].

Our patient was 36 weeks premature and presented in the NICU after spontaneous vaginal delivery to consanguineous marriage because of the unusual appearance of the baby that was not apparent on previous ultrasounds. Peripheral Cyanosis was present, and the baby had severe scalded skin with deep fissures covering most of the body parts, along with distorted facial features and tight skin around the mouth, which restricted feeding, and around the chest, which restricted respiration. Electrolyte imbalance and Dehydration were apparent. The patient was treated with a combination of prophylactic antibiotics, early administration of oral retinoids, and topical eye drops to prevent eye infection. Petroleum jelly was applied to help with the shedding of defective, hard skin. Supplemental oxygen with nasal prongs and electrolyte replenishment was done. No surgical intervention was performed. Significant improvement was noted after initial therapy, but unfortunately, the patient did not survive beyond the ninth day of life.

## Conclusions

In conclusion, this case report provides valuable insights into the clinical presentation, diagnosis, and management of Harlequin ichthyosis. It underscores the importance of a comprehensive, multidisciplinary approach to address the complex needs of patients with this condition. Further research and collaboration are needed to deepen our understanding of Harlequin ichthyosis and develop more targeted therapeutic interventions, ultimately improving outcomes and quality of life for those affected by this rare genetic anomaly.
